# Supplementary material for: Regulation of INSM1 Gene Expression and Neuroendocrine Differentiation in High-Risk Neuroblastoma
Source: Biology (Basel). 2025 Dec 22;15(1):22. doi: 10.3390/biology15010022 (PMC12784908; doi:10.3390/biology15010022)
Supplement: Supplementary file 1 [file biology-15-00022-s001.zip › biology-4013292-supplementary.pdf]

Figure S1

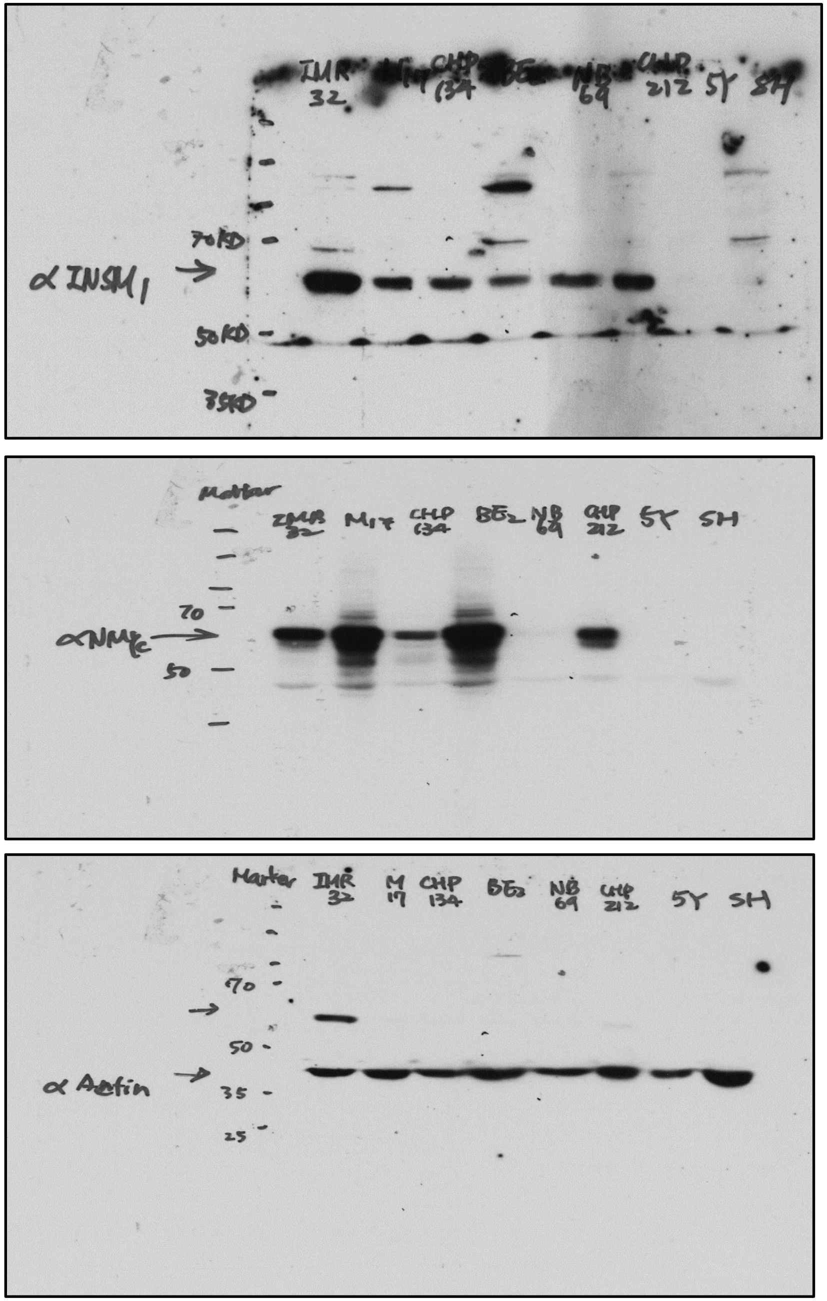
IMR 32
CHP 134
NB 69
CHP 212
5Y
70KD
α INSM1
50KD
35KD
Marker
αNMyc
70
50
M 17
BE2
SH
α Actin
35
25

Figure S2

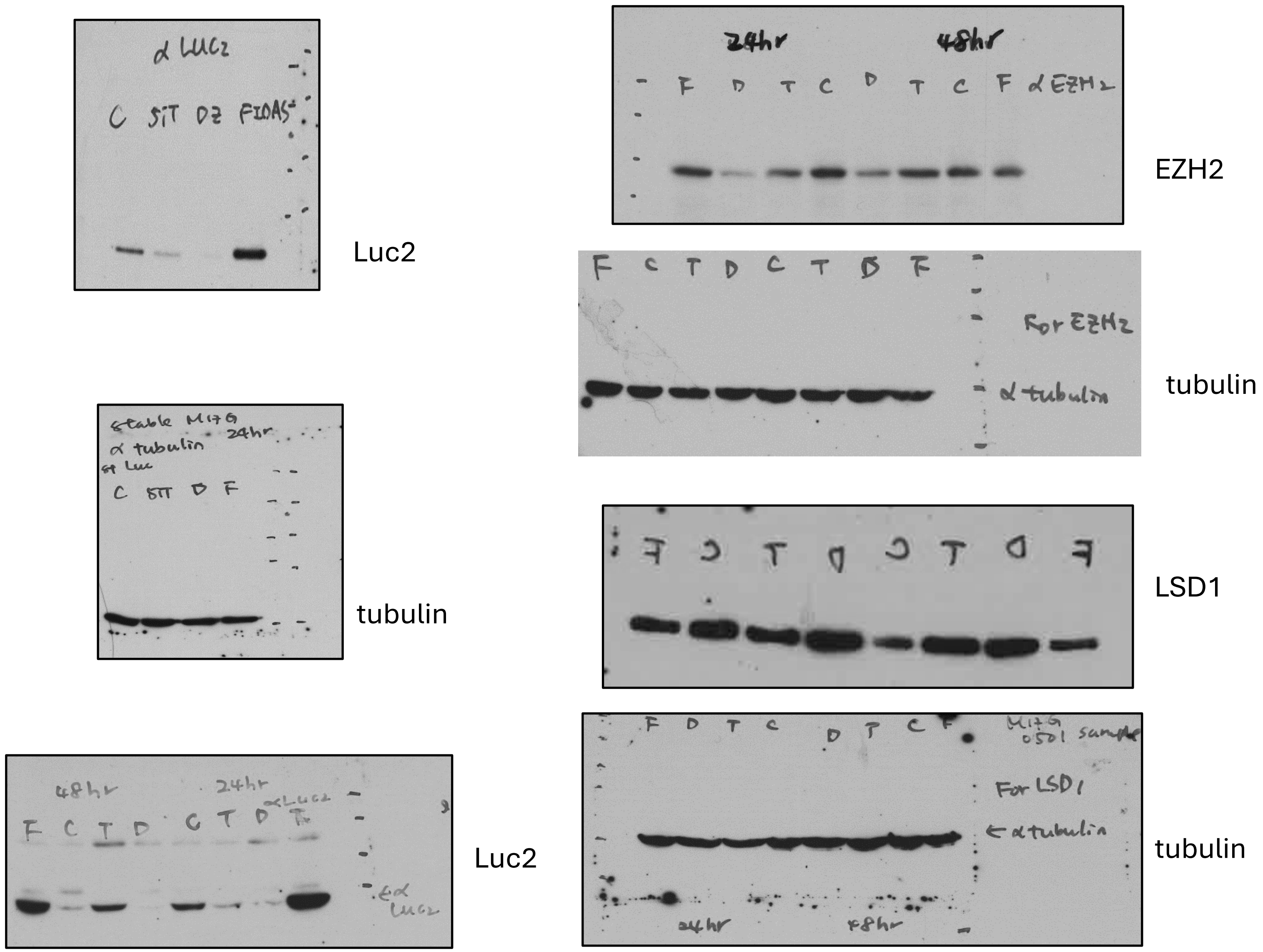

Figure S3

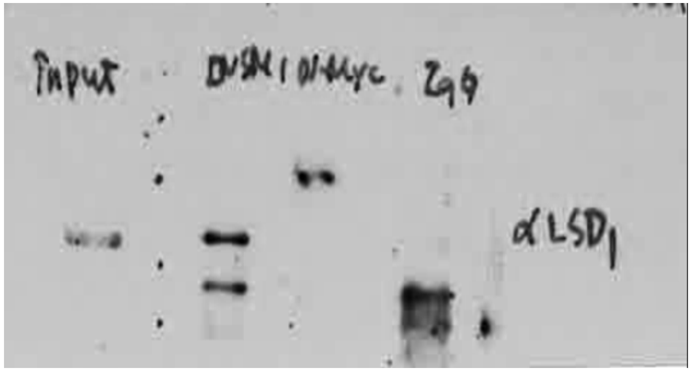

Co-IP

Figure S4

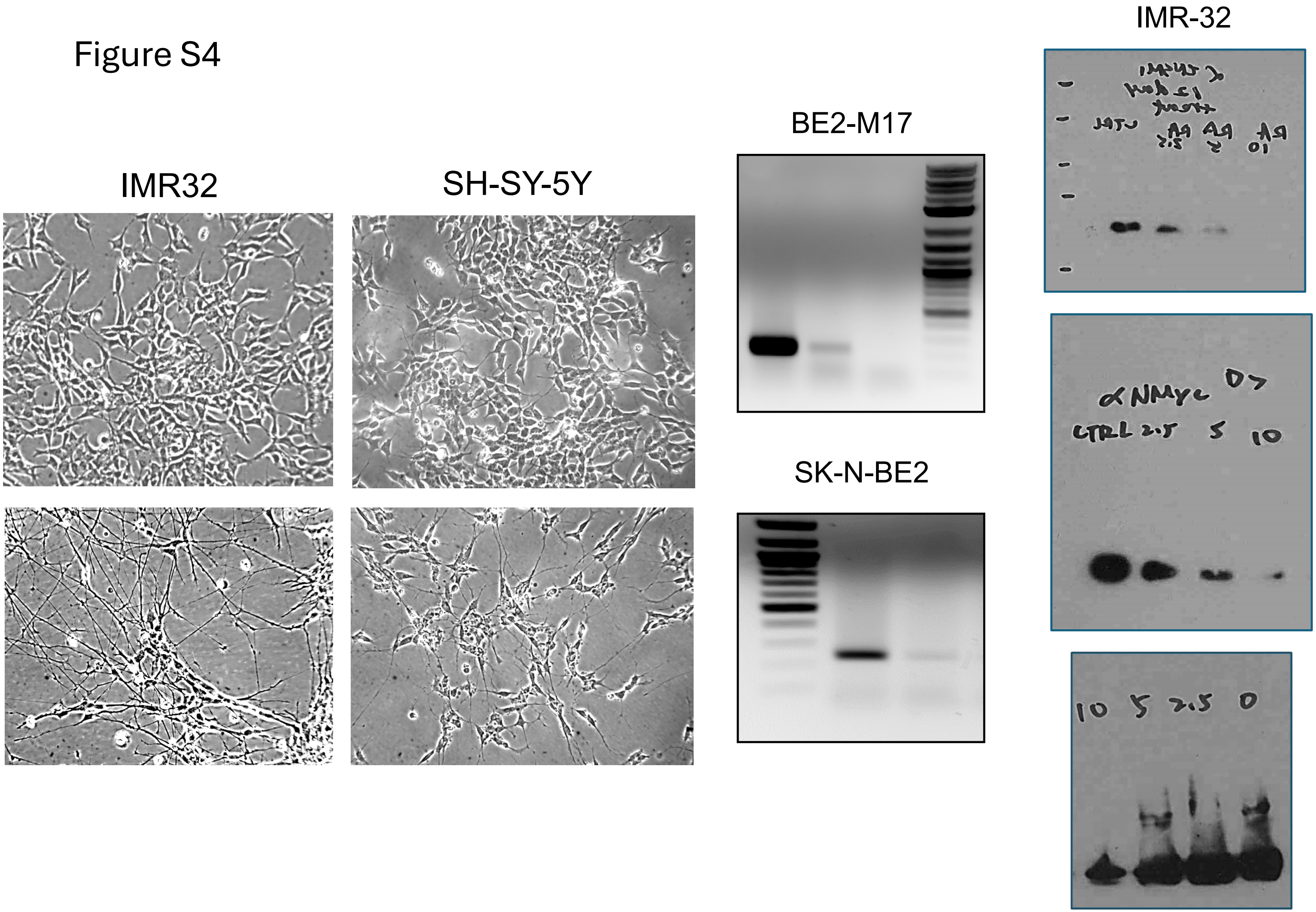
IMR32
SH-SY-5Y
BE2-M17
SK-N-BE2
IMR-32
αNMyc D7
CTRL 2.5 5 10
10 5 2.5 0
